# Effect of Metaldehyde on Survival, Enzyme Activities, and Histopathology of the Apple Snail *Pomacea canaliculata* (Lamarck 1822)

**DOI:** 10.3390/biology13060428

**Published:** 2024-06-11

**Authors:** Jimin Liu, Xuan Chen, Jiaen Zhang, Fucheng Yao, Zhaoji Shi, Yingtong Chen, Qi Chen, Zhong Qin

**Affiliations:** 1 Department of Ecology, College of Natural Resources and Environment, South China Agricultural University, Guangzhou 510642, China; liujimin1213@163.com (J.L.); 18819265935@163.com (X.C.); yaofuch@163.com (F.Y.); shizhaojiyyy@gmail.com (Z.S.); yingtongchenchn@163.com (Y.C.); qiqichen0913@163.com (Q.C.); 2 Guangdong Provincial Key Laboratory of Eco-Circular Agriculture, South China Agricultural University, Guangzhou 510642, China; 3 Guangdong Engineering Research Center for Modern Eco-Agriculture and Circular Agriculture, Guangzhou 510642, China; 4 Key Laboratory of Agro-Environment in the Tropics, Ministry of Agriculture and Rural Affairs, Guangzhou 510642, China

**Keywords:** molluscicide, digestive gland, acute toxicity, histopathology

## Abstract

**Simple Summary:**

The invasion of the invasive alien species *Pomacea canaliculata* is of great concern because of its adverse influences on ecosystems, agriculture, and human health in the Asian region. Metaldehyde-containing molluscicides are often used as an effective method of controlling this species. In view of this, the present study conducted experiments to investigate the lethal concentrations of metaldehyde on *Pomacea canaliculata* at different exposure times. Moreover, the study also elucidated changes in enzyme activity and provided detailed histopathological evidence that shed light on the mode of action and effects of metaldehyde exposure on *Pomacea canaliculata*. The findings of this study not only deepen our understanding of the effects of metaldehyde on mollusks, but also provide important insights for developing better control strategies.

**Abstract:**

*Pomacea canaliculata*, as an invasive exotic species in Asia, can adversely affect crop yields, eco-environment, and human health. Application of molluscicides containing metaldehyde is one effective method for controlling *P. canaliculata*. In order to investigate the effects of metaldehyde on adult snails, we conducted acute toxicological experiments to investigate the changes in enzyme activities and histopathology after 24 h and 48 h of metaldehyde action. The results showed that the median lethal concentrations (LC) of metaldehyde on *P. canaliculata* were 3.792, 2.195, 1.833, and 1.706 mg/L at exposure times of 24, 48, 72, and 96 h, respectively. Treatment and time significantly affected acetylcholinesterase (AChE), glutathione S-transferase (GST), and total antioxidant capacity (TAC) activity, with sex significantly affecting AChE, GST, and TAC activity and time significantly affecting carboxylesterase (CarE). In addition, the interaction of treatment and time significantly affected the activity of GST, CarE and TAC. In addition, histopathological changes occurred in the digestive glands, gills and gastropods of apple snail exposed to metaldehyde. Histological examination of the digestive glands included atrophy of the digestive cells, widening of the hemolymph gap, and an increase in basophils. In treated snails, the hemolymph gap in the gills was widely dilated, the columnar cells were disorganized or even necrotic, and the columnar muscle cells in the ventral foot were loosely arranged and the muscle fibers reduced. The findings of this study can provide some references for controlling the toxicity mechanism of invasive species.

## 1. Introduction

*Pomacea canaliculata* (Lamarck 1822), a freshwater gastropod native to South America, is presently broadly conveyed in tropical and subtropical locales [1]. It was introduced to Asia in the early 1980s as a food source for humans [2]. Apple snails are highly adaptable, fast growing and fast reproducing [3,4]. They have caused serious damage and impact on aquatic crops (e.g., rice, vegetables) and eco-environment in the invaded area [5,6,7,8].

Invasive apple snails, *P. canaliculata* and *Pomacea* maculate (Perry, 1810), [formerly *P. insularum* (d’Orbigny, 1835)] (Caenogastropoda: Ampullariidae) have a widespread distribution globally [9]. Despite the long-standing belief that only *P. canaliculata* existed as an exotic *Pomacea* species in Asia due to morphological similarities [10], molecular methods since 2010 have confirmed the coexistence of *P. canaliculata* and *P. maculata* populations in China [11]. Because *Pomacea* snails are highly adaptable and tolerant, they have established populations in 16 provinces and regions of China and are expected to spread further north [12]. Molluscicides are the highly effective and widely used method to control snail [13]. Metaldehyde, the main active ingredient in molluscicides, is used worldwide to control most slugs and snails [14]. Widely employed since its introduction as a molluscicide in 1936, metaldehyde’s mobility in soil and groundwater contamination have raised concerns regarding its environmental impact [15,16,17,18]. In addition, metaldehyde causes mortality in non-target organisms, including vertebrates and humans, suggesting a wide range of ecological impacts [19,20].

The exact mechanism by which metaldehyde causes the death of snails is still unknown [21]. It is often described as causing permanent damage to molluscan epithelial cells, leading to molluscan death, but its mode of action has rarely been studied [22]. The effect of pesticides on organisms can also be explained by limiting the detoxification capacity of certain enzymes by inhibiting their activity [23]. Notably, Zhang et al. [24] observed fluctuations in acetylcholinesterase (AChE) and glutathione s-transferase (GST) activities in *P. canaliculata* in response to sublethal metaldehyde exposure, highlighting the dynamic nature of the response to pesticide stress in *P. canaliculata*. Arrighetti et al. [25] found some significant differences between male and female snails in some of the biomarkers analyzed, but it is not clear if they are related to different sensitivities to exposure to toxic effects. Histopathological tests are useful tools to characterize toxic substances in living organisms [26]. Histopathological assessments reflect the cellular and tissue alterations caused by toxic substances and provide valuable information for understanding the effects of toxicants on organisms [27,28].

In the current research, survival, enzyme activities and tissue structure in snails was investigated though an acute toxicological experiment, in addition to analyzing the differences in these biomarkers between males and females with the aim of evaluating the toxic effects of metallic aldehydes on snails. Our results help to understand the physiological functions of snails under pesticide stress, and also the mechanisms of toxicity in controlling invasive species, and provide some references for farming practices.

## 2. Materials and Methods

### 2.1. Snail Collection

Snails were firstly collected in April 2022 in Kaizhou District, Chongqing city, China (31°26′ N, 108°43′ E). After that, in order to obtain pure *P. canaliculata*, we placed the snails on the Teaching and Research Farm of South China Agricultural University (23°9′ N, 113°21′ E) for separating female and male pair breeding, and collected egg masses from each family. Amplification of the mitochondrial Cytochrome c oxidase subunit I gene (COI) using primers LCO1490 and HCO2198 was performed to differentiate snail species [1,29]. To ensure that only *P. canaliculata* were tested, egg masses of *P. canaliculata* were selected for hatching and then fed with fresh lettuce (*Lactuca sativa* L.) until adulthood (shell diameter 25–35 mm) in a pond (3 m × 1 m × 1 m) that had not been exposed to pesticides. After that, these pure snails were collected for testing in the laboratory, and acclimated in a 100-L (650 mm × 480 mm × 350 mm) aquarium at 26 ± 2 °C and pH 7.25 ± 0.25 for 10 days. During acclimation, two-thirds of the dechlorinated tap water was replaced daily and fed with the equivalent of the body weight of lettuce. No feedings were supplied for the snails for 24 h before the measurement and during the exposure period.

### 2.2. Experimental Design

Metaldehyde (CAS: 108-62-3), containing 40% of the active ingredient of metaldehyde was used and purchased from Nongmi Biotechnology Co., Ltd. (Guangzhou China).

Five concentrations (1.29, 2.00, 3.12, 4.87, and 7.59 mg/L) were prepared for the exposure experiments. The metaldehyde stock solution was obtained by dissolving metaldehyde in analytically pure-grade dimethyl sulfoxide [DMSO, Jiangsu Qiangsheng Functional Chemical Co., Ltd.] (Nanjing China) and sonicating [22,30]. Serial dilutions were then made to obtain the desired concentration. Tests were conducted in a 100-L aquarium which was covered with clip-fastened gauze overhead to prevent snails from escaping. Thirty snails, 15 males and 15 females, were used for each concentration treatments and control, and each exposure experiment was repeated three times. During the 96-h exposure period, the test solution was renewed every 24 h, and the number of dead snails was recorded [31]. Dead individuals were removed promptly during the test, in which snails were considered dead when they did not contract and did not make any movement when the operculum nail was lightly touched with forceps. No food was provided.

### 2.3. Biochemical Analysis

Four doses were set at 1.896, 0.948, 1.098, and 0.549 mg/L for 1/2 and 1/4 of the Median lethal concentration (LC_50_) at 24 h and 48 h, respectively. After 24 h and 48 h of exposure, digestive glands were quickly dissected from the shell and weighed, mixed with saline (0.9%) at a ratio of 1:9 (*w*/*v*), and homogenized in a tissue grinder (Tissuelyser-32 L) for 60 s. To determine the levels of biochemical parameters, an ice-cold refrigerated centrifuge was used to centrifuge the supernatant at 12,000 rpm for 30 min. AChE activity was measured by the increase in yellow color produced by the reaction of thiocholine with dithiodinitrobenzoic acid ions [32]. GST activity was measured by the change in substrate concentration before and after the reaction of reduced Glutathione (GSH) with the substrate 1-chloro-2,4 dinitrobenzene (CDNB) [33]. Carboxylesterase (CarE) activity was determined by measuring the rate of hydrolysis of 1-naphthyl acetate (1-NA) as a substrate [34]. Total antioxidant capacity (TAC) was determined by enzymatic reaction using the colourimetric method [35]. AChE, TAC and GST were measured with a microplate spectrophotometer [BioTek Epoch] (Guangzhou, China) and CarE was measured with a UV-visible spectrophotometer (T-UV1810). The above enzyme activities were assayed with kits purchased from the Nanjing Jiancheng Institute of Biological Engineering (Nanjing, China).

### 2.4. Histological Preparation

After 24 and 48 h of exposure to the test solution, the digestive glands, gills, and gastropods of *P. canaliculata* were removed and preserved in Paraformaldehyde Fixation (4% PFA). Each treatment was replicated four times with five tablets per replication, and dechlorinated tap water was used as a control. Samples were dehydrated in an ascending ethanol series, then cleared with xylene and embedded in paraffin, cut to a thickness of 4–6 μm, and sections mounted on slides were stained using Harris hematoxylin and eosin. Histopathological changes in the sections were examined using a microscope (Leica DM2000 LED). Histopathological alterations were described and categorized according to the frequency of occurrence of such alterations, with the following criteria: “-” = none (no slides presenting alterations), “+” = mild (<25% of slides analyzed), “++” = moderate (alterations appearing in 25–75% of slides) and “+++” = severe (alterations appearing in >75% of slides analyzed) [36].

### 2.5. Data Analysis

Statistical analyses were performed using SPSS 26.0 (IBM Corporation, New York, NY, USA). Lethal concentration (LC) was obtained by using probabilistic analysis, dose–response data analysis; Comparative analysis of enzyme activities was performed using three-way ANOVA with a significance level of 5%. Graphs were prepared using software R (4.3.3).

## 3. Results

### 3.1. Snail Activity

In acute toxicology experiments, no snails died in the control group, while the other treatment groups showed varying levels of toxicity, as evidenced by the secretion of large amounts of clear mucus, incapacitation, and the post-mortem closure of the operculum or exposure of the whitish gastropods. The LC_50_ values were 3.792, 2.195, 1.833, and 1.706 mg/L after 24, 48, 72, and 96 h of treatment, respectively (Table 1).

### 3.2. Enzyme Activities

**Figure 1:**
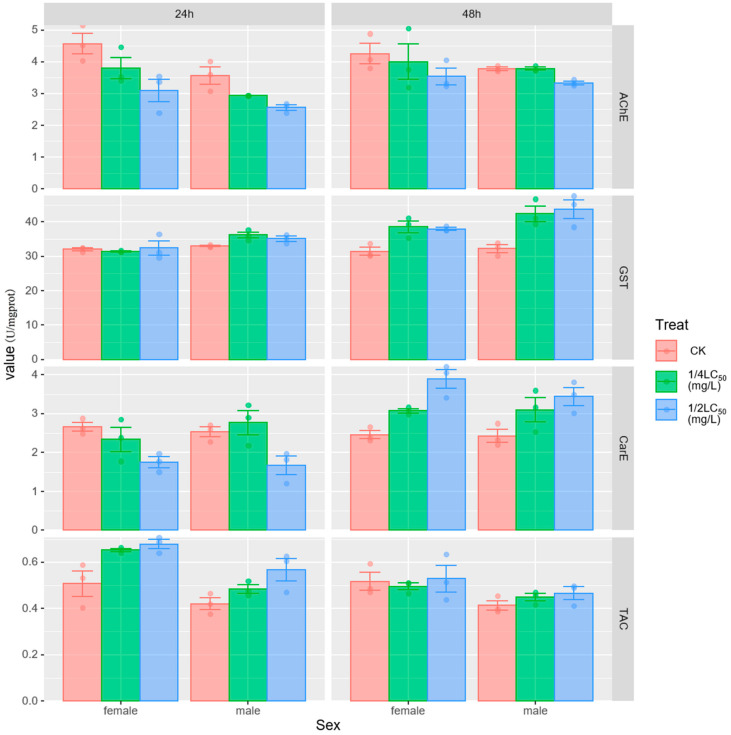
Effect of sublethal concentrations of metaldehyde on the enzymatic activities of AChE, GST, CarE and TAC in the digestive gland of *P. canaliculata.* CK indicates control; data are expressed as mean ± SE.

The impact of metaldehyde on the digestive gland enzyme activity of *P. canaliculata* is depicted in Figure 1. Three-way ANOVA results showed that the treatment (all *p* < 0.01) and time (all *p* < 0.05) significantly affected AChE, GST, and total antioxidant capacity (TAC) activities; sex significantly affected AChE (*p* = 0.002), GST (*p* < 0.001), and TAC (*p* < 0.001) activities; time significantly affected CarE (*p* < 0.001); AChE and TAC activities were not affected by interaction between the treatment, time, and sex; AChE and TAC activities were unaffected by the interaction between the treatment, time, and sex; and GST and CarE activities were unaffected by the interaction of sex with time and treatment. In addition, the interaction of the treatment and time significantly affected the activities of GST (*p* < 0.001), CarE (*p* < 0.001), and TAC (*p* < 0.05) (Table 2). Specifically, the treatment caused a decrease in AChE activity at both 24 and 48 h, especially at the 1/2 LC_50_ concentration. And AChE enzyme activity was lower in male snails compared with female snails. The treatment increased GST activity, especially at 48 h. And GST enzyme activity was higher in male snails compared with female snails. This may be due to the processing-timing interaction. The treatment decreased CarE activity at 24 h, increased CarE activity at 48 h, and increased CarE activity at 48 h compared with control. The treatment increased TAC activity at 24 h and 48 h. Only female snails showed a decrease after 48 h in the 1/4 LC_50_ concentration, and TAC activity was higher at 24 h than at 48 h. The treatment also increased TAC activity at 24 h compared with the control.

### 3.3. Histopathology

#### 3.3.1. Histopathological Response of the Digestive Glands

**Figure 2:**
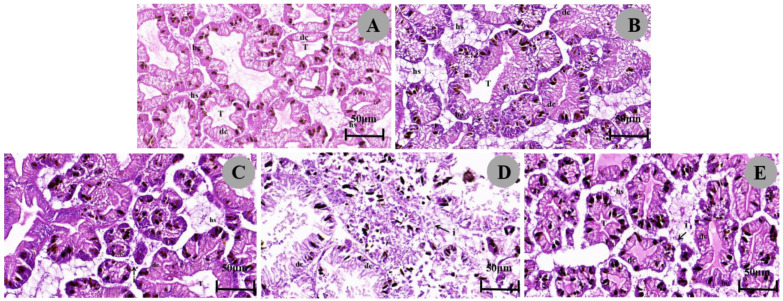
Micrographs of *P. canaliculata* digestive glands. (**A**). Digestive glands of control snails showing digestive cells (dc), basophils (bc), tubular lumen (T), and dark granules (dg). (**D**,**E**). digestive glands were exposed to 1/2 LC_50_ metaldehyde. (**B**,**C**). Digestive glands exposed to 1/4 LC_50_ metaldehyde show a widening of the hemolymph gap and an increase in basophils.

The digestive gland of *P. canaliculata* consisted of many irregular round structures, including many digestive cells and some blind-terminated tubules composed of basophilic cells, with hemolymph gaps between the tubules (Figure 2A; Table 3). The digestive cells were columnar, and the cytoplasm contained granules of different sizes that were faintly stained and the nucleus was pushed towards the basolateral membrane. Basophils were triangular in shape and appeared significantly less frequently than digestive cells, in which some oval, dark granules appeared. Under 1/4 LC_50_ treatment after 48 h of exposure, the hemolymph gap widened and increased (Figure 2E), and under 1/4 LC_50_ treatment after 24 h of exposure, the digestive cells atrophied, the hemolymph gap widened, and inflammatory cell infiltration occurred with an increase in basophils (Figure 2C). Under 1/2 LC_50_ treatment, the digestive cells were more severely atrophied or even necrotic, with a marked increase in dark granules, a widened hemolymph gap, and an incomplete digestive gland structure (Figure 2B,D).

#### 3.3.2. Histopathological Reaction of the Gills

**Figure 3:**
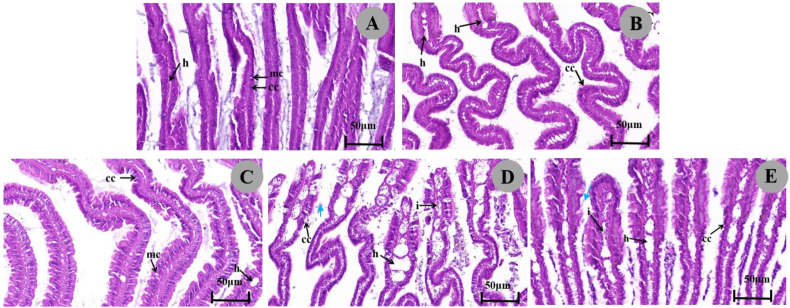
Micrographs of the gills of *P. canaliculata*. (**A**). In details of gill filaments of control group snails, normal gill filament columnar cells (CC) are tightly arranged, a few mucous cells (MC) are visible, and the hemolysis gap (h) is narrow. (**D**,**E**). Gill filaments exposed to 1/2 LC_50_ of metaldehyde show disorganized gill filament columnar cells (CC) with cell degeneration, cilia loss (blue arrows), a greatly expanded hemolysis gap (h), and inflammatory cell infiltration (i). (**B**,**C**). Gill filaments exposed to 1/4 LC_50_ metaldehyde show slight loosening and degenerative arrangement of gill filament columnar epithelial cells (CC) and more area of hemolysis gap expansion (h).

Histological sections of gills in control group were observed, and the results showed that the gills of *P. canaliculata* consisted of gill filaments with an epithelium containing well-arranged cilia, ciliated columnar cells, and mucous cells. The ciliated columnar cells of the gill tissue as well as the narrow hemolymph gap were structurally normal (Figure 3A; Table 3). After exposure to an LC_50_ concentration of 1/4, columnar cells were slightly loosened and degenerated in arrangement, and the area of the hemolymph gap was expanded (Figure 3C,E). Under 1/2 LC_50_ treatment, columnar cells were disorganized, even necrotic, detached, and accompanied by inflammatory cell infiltration, and the hemolymph gap was severely dilated over a large area (Figure 3B,D). It is noteworthy that these symptoms worsened with increasing concentration of metaldehyde.

#### 3.3.3. Histopathological Response of the Ventral Foot

**Figure 4:**
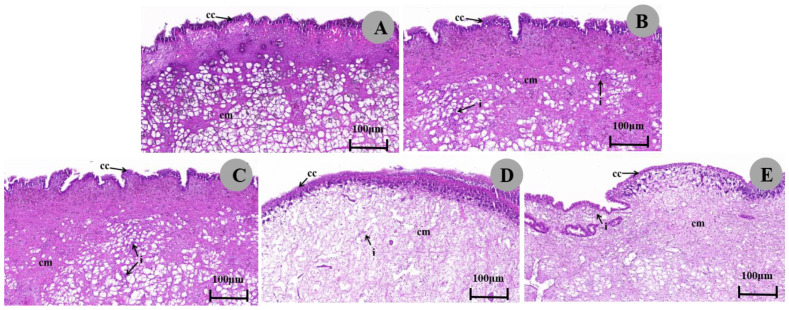
Micrographs of the ventral foot of *P. canaliculata*. (**A**). The ventral foot of the control group snails shows a neat and orderly arrangement of epithelial cells (e), normal morphology of columnar muscle cells (cm), and an interlocking arrangement of muscle fibers. (**D**,**E**). The ventral foot of the snail exposed to 1/2 LC_50_ metaldehyde shows degeneration, necrosis, and an indistinct morphology of the epithelial cells (e) of the ventral foot. Columnar muscle cells (cm) were loosely arranged and scattered, with a marked reduction in myofibers and scattered interstitial inflammatory cell infiltration (i). (**B**,**C**). The ventral foot of snails exposed to 1/4 LC_50_ metaldehyde shows varying degrees of inflammatory cell infiltration visible between fibers (i).

The ventral foot tissue of the control group contained mainly epithelial cells and columnar muscle cells (Figure 4A; Table 3). Compared with the control, after 1/4 LC50 exposure, the epithelial cells of the ventral foot were neatly arranged and orderly, the columnar muscle cells were morphologically normal, and different degrees of inflammatory cell infiltration was seen between the fibers (Figure 4C,E). Under 1/2 LC_50_ treatment, ventral foot epithelial cells became short, necrotic, and morphologically indistinct; columnar muscle cells were loosely arranged and scattered; muscle fibers were reduced to varying degrees; interstitial scattered inflammatory cell infiltration was observed (Figure 4B,D). Again, these symptoms worsened with increasing concentration of metaldehyde.

## 4. Discussion

### 4.1. Effect of Metaldehyde on Enzyme Activity

AChE is an enzyme involved in the termination of nerve impulse transmission by catalyzing the hydrolysis of the neurotransmitter acetylcholine (ACh) [37]. Metaldehyde poisoning disrupts acetylcholine binding to the receptor, leading to changes in acetylcholine activity, which in turn affects behavior [38]. The AChE activity in the soft tissues of freshwater snails was significantly inhibited by the fungicide avermectin and gradually recovered over time [39]. We observed that AChE activity in the digestive gland of *P. canaliculata* was significantly suppressed after exposure to metaldehyde, and AChE activity was lower in male snails compared with females, which may be due to the fact that metaldehyde affected the accumulation of ACh in the synaptic gap, leading to paralysis of the postsynaptic membrane and neurological dysfunction [40], which ultimately resulted in the weakening of the snail’s locomotor and feeding behaviors and even to death, suggesting that the behaviour of male snails would be more severely affected. The main organ for detoxification in mollusks in stages I and II is the digestive gland. When phase I enzymes are unable to cope with toxic substances and cells are damaged, GST activity increases and promotes redox homeostasis, playing a key detoxification role, which is particularly evident in the liver [41,42]. Several studies have shown that GST activity in the digestive glands of snails increased when exposed to pesticides or pollutants. Studies found that cadmium increased GST activity in the digestive glands of *P. canaliculata* [43]. This is consistent with our findings that the treatment increased GST, especially at 48 h, and that male snails had higher GST enzyme activity compared with females. Increased GST activity may contribute to cellular redox homeostasis and protect cells from oxidative damage [44], suggesting that female snails are more susceptible to oxidative damage. CarE consists of various isoenzymes that alter tissues and organisms, and these enzymes are important in the metabolic and detoxification processes of mollusks [45]. We found that CarE activity in snails with 1-NA as substrate decreased significantly after 24 h and increased significantly after 48 h, and that CarE activity was higher at 48 h than at 24 h. Similarly, Cacciatore [46] observed that azinphos-methyl (AZM) and chlorpyrifos (CPF) highly inhibit the CarE activity of *Planorbarius corneus* (*Gastropoda*: *Planorbidae*) snails. This may be due to the activation of snail metabolism by metaldehyde. TAC represents the combined antioxidant activity of all antioxidants present in biological samples, including both enzymatic and non-enzymatic antioxidants, and the determination of TAC has been widely used as a tool for evaluating the state of redox reactions and the level of total antioxidant capacity in organisms [47]. Our results found that the treatment increased TAC activity at 24 and 48 h, only female spirochetes decreased after 48 h in the 1/4 LC_50_ concentration, and TAC activity was higher at 24 h than at 48 h. This is in line with the findings of Cossi et al. [48] who found that TAC activity in *Biomphalaria straminea* increases under the influence of organophosphorus insecticides. Total antioxidant capacity represents the antioxidant ability within the organism [49]. The increase in TAC may be due to induction of antioxidant defences and, conversely, the decrease in TAC levels can be attributed to depletion of antioxidant defences and the onset of oxidative stress [50]. Thus, it is clear that the total antioxidant capacity of *P. canaliculata* plays an important role in resistance to metaldehyde toxicity.

Interestingly, sex significantly affected AChE, TAC, and GST activities in this study, but sex did not significantly affect enzyme activities in interaction with time or treatment. Differences also existed between some biochemical parameters in the digestive gland of *P. canaliculata* of different sexes exposed to cypermethrin (CYP) [25].

### 4.2. Effect of Metaldehyde on the Histopathology

Histological observations of this study showed that metaldehyde exposure resulted in structural and morphological changes in the tissues of the digestive glands, gills, and gastropods of *P. canaliculata*. These changes may affect the viability and ecological functions of the snail, with potential implications for the stability of aquatic ecosystems.

One of the most important organs in mollusks that are attacked by toxic substances is the digestive gland. We found that metaldehyde treatments at different concentrations and duration times caused different degrees of damage to the digestive glands. The digestive gland of *P. canaliculata* showed atrophy of digestive cells, an increase in the number of basophils, and a greater abundance of digestive cells than basophils under stress conditions, probably due to the loss of digestive cells resulting in the increased relative number of basophils [51]. The increase in dark particles indicated the accumulation of toxic substances such as molluscicides [52]. They also included the widening of the hemolymph gap and inflammatory cell infiltration. Similarly, *P. canaliculata* exposed to sublethal copper sulfate [53] and *Marisa cornuarietis* (*Gastropoda*: *Prosobranchia*) exposed to copper and lithium [54] showed the same changes in their digestive glands. These changes are dose-dependent, in addition, high concentrations of metaldehyde can disrupt the structure of the digestive glands, possibly affecting biochemical pathways and ultimately leading to the death of *P. canaliculata*.

During metaldehyde exposure, we observed that snails moved only underwater or at rest, so the gills may be the organ that frequently contacts directly with the pesticide during metaldehyde treatments. Gills are an important organ in aquatic organisms regarding oxygen uptake, so increased mucus production may be the first response to mechanically protect the epithelium [55]. Damage to gill tissue included reduced or absent cilia length and increased numbers of mucus cells, which may cause reduced oxygen consumption and disruption of osmoregulatory processes. This is consistent with histological changes in *P. canaliculata* exposed to contaminants, suggesting that these symptoms are non-specific [56].

The histopathological changes in the gastropods of *P. canaliculata* were mainly characterized by epithelial cell degeneration and necrosis and a marked reduction in myofibers. The foot of *Monacha obstructad* (*Gastropoda*: *Hygromiidae*) snails treated with LC_50_ of Jatropha showed rupture of the epithelium covering the foot, desquamation of the epithelium and presence of areas of connective tissue necrosis and destruction of the muscular tissue [57]. This is consistent with our observations. *P. canaliculata* has a feeding pattern that utilizes the surface of its feet to generate pedal waves that float across the air–water interface to collect food. Thus, dissolution of muscle fibers in the feet may cause disruption of feeding behavior [43].

## 5. Conclusions

In the present study, the toxic effects of metaldehyde on *P. canaliculata* were thoroughly investigated, and some important findings were made. The toxic effects of metaldehyde caused oxidative damage and activated the antioxidant system of *P. canaliculata*, which was able to improve its survival capacity by regulating the levels of physiological and biochemical substances in the body. Histological changes included the appearance of digestive gland with atrophy of digestive cells and increase in basophils, and gill tissues showed slight to severe damage including loosening, necrosis, and detachment of gill filament columnar epithelial cells, haemolymphatic interstitial dilatation and other pathological damage, and the ventral foot tissues showed epithelial cell degeneration, necrosis and reduction in muscle fibres, which ultimately affected their viability. These findings have important implications for agricultural practices and ecosystem management. Understanding the mechanism of metaldehyde toxicity can help us better evaluate and manage its use in agricultural production, thereby reducing the impacts on non-target organisms and preserving ecosystem stability.

Future research should focus on exploring the behavior and population dynamics of *P. canaliculata*, and ecosystem health under long-term exposure to metaldehyde, using an integrated interdisciplinary approach. In addition, elucidating the molecular mechanisms of metaldehyde toxicity and sex-specific responses of *P. canaliculata* to the toxicity are also important directions for future research.

## Figures and Tables

**Table 1:** LC_50_ values of *Pomacea canaliculata* exposed to metaldehyde for 24 h, 48 h, 72 h, and 96 h, with confidence limits of 95%.

Time (h)	LC_50_ (mg/L)	χ^2^	r	95% Confidence Limits
24	3.792	11.957	0.968	3.516–4.103
48	2.195	12.588	0.943	2.012–2.378
72	1.833	12.923	0.909	1.661–1.997
96	1.706	15.929	0.867	1.560–1.842

**Table 2:** Sum of squares (SS), degrees of freedom (df), mean square residuals (MS), Fisher’s test (F) and *p*-value (*p*) of the multifactorial ANOVA for the effect of the treatments, time and sex and their interactions on enzyme activity.

	Factors	df	SS	MS	F	*p*
AChE	Treat	2	4.97	2.48	10.94	<0.01
	Time	1	1.18	1.18	5.18	0.03
	Sex	1	2.76	2.76	12.14	<0.01
	Treat × Time	2	0.76	0.38	1.66	0.21
	Treat × Sex	2	0.21	0.11	0.47	0.63
	Time × Sex	1	0.58	0.58	2.56	0.12
	Treat × Time × Sex	2	0.04	0.02	0.09	0.92
GST	Treat	2	202.92	101.46	17.09	<0.01
	Time	1	171.76	171.76	28.94	<0.01
	Sex	1	87.24	87.24	14.70	<0.01
	Treat × Time	2	114.41	57.21	9.64	<0.01
	Treat × Sex	2	23.54	11.77	1.98	0.16
	Time × Sex	1	0.88	0.88	0.15	0.70
	Treat × Time × Sex	2	7.24	3.62	0.61	0.55
CarE	Treat	2	0.54	0.27	1.95	0.16
	Time	1	5.44	5.44	39.60	<0.01
	Sex	1	0.01	0.01	0.10	0.75
	Treat × Time	2	6.97	3.48	25.36	<0.01
	Treat × Sex	2	0.38	0.19	1.38	0.27
	Time × Sex	1	0.12	0.12	0.84	0.37
	Treat × Time × Sex	2	0.12	0.06	0.45	0.65
TAC	Treat	2	0.06	0.03	8.25	<0.01
	Time	1	0.05	0.05	14.52	<0.01
	Sex	1	0.08	0.08	25.129	<0.01
	Treat × Time	2	0.03	0.01	3.881	0.03
	Treat × Sex	2	0.0006	0.0003	0.089	0.91
	Time × Sex	1	0.006	0.006	1.737	0.20
	Treat × Time × Sex	2	0.007	0.004	1.058	0.36

**Table 3:** Effect of metaldehyde on the histomorphology of *P. canaliculata*.

Tissues	Histopathological Effects	Control	1/2 LC_50_ (24 h)	1/4 LC_50_ (24 h)	1/2 LC_50_ (48 h)	1/4 LC_50_ (48 h)
Digestive gland	Number of basophils	-	++	+	+	+
	Digestive cell atrophy	-	++	++	++	++
	Expansion of the hemolymphatic space	-	+	+	+	+
Gill	Ciliate shedding	-	++	-	+	-
	Columnar cell changes	-	+++	-	++	+
Ventral foot	Epithelial cell atrophy	-	+	-	++	-
	Columnar muscle cell dispersion	-	++	-	+	-
	Decrease in muscle fibers	-	+++	-	++	-

Note: Score value: -: no histopathology, +: mild histopathology (present in <25% of the slides), ++: moderate histopathology (present in 25–75% of the slides) and +++ = severe histopathology (present in >75% of the slides).

## Data Availability

Dataset available on request from the authors.
